# Introgression of null allele of Kunitz trypsin inhibitor through marker-assisted backcross breeding in soybean (*Glycine max* L. Merr.)

**DOI:** 10.1186/s12863-016-0413-2

**Published:** 2016-07-12

**Authors:** Shivakumar Maranna, Khushbu Verma, Akshay Talukdar, Sanjay Kumar Lal, Anil Kumar, Keya Mukherjee

**Affiliations:** ICAR- Indian Institute of Soybean Research, Indore, MP India; Division of Genetics, ICAR-Indian Agricultural Research Institute, New Delhi, 110012 India

**Keywords:** Foreground and background selection, Kunitz trypsin inhibitor, Marker-assisted backcross breeding, Null allele, SSR markers

## Abstract

**Background:**

Presence of Kunitz trypsin inhibitor (KTI) in soybean seeds necessitates pre-heat treatment of the soy-flour for its inactivation before using it in food and feed products. The heat treatment not only enhances processing costs of the soy-based foods and feeds but also affects seed-protein quality and solubility. Genetic elimination of KTI is an important and effective approach. Therefore, molecular marker-assisted backcross breeding (MABB) approach was adopted for genetic elimination of KTI from two popular soybean genotypes, DS9712 and DS9814. PI542044, an exotic germplasm line was used as donor of the *kti* allele which inhibits functional KTI peptide production.

**Results:**

Foreground selection for the *kti* allele was performed with three closely linked SSR markers while background selection was done with 93 polymorphic SSR markers. Plants in the BC_1_F_1_ generation were found to recover 70.4–87.63 % and 60.26–73.78 % of the recurrent parent genome (RPG) of DS9712 and DS9814, respectively. Similarly, selected plants in the BC_2_F_1_ generation had 93.01–98.92 % and 83.3–91.67 % recovery of their respective RPGs. Recombinant selection was performed so as to identify plants with minimal linkage drag. Biochemical analysis of the seeds of the selected plants (*ktikti*) confirmed absence of KTI peptides in the seeds. Phenotypically, the selected plants were comparable to the respective recurrent parent in yield and other traits.

**Conclusions:**

MABB approach helped in speedy development of 6 KTI free breeding lines of soybean. Such lines will be suitable for the farmers and the soybean industries to use in production of soy-based foods and feeds without pre-heat treatment of the soy-flour. It would contribute towards wider acceptability of soy-based foods and feeds.

**Electronic supplementary material:**

The online version of this article (doi:10.1186/s12863-016-0413-2) contains supplementary material, which is available to authorized users.

## Background

Soybean is an important source of high quality oil and protein for both human and animal. However, it cannot be fed raw or unprocessed to the mono-gastric animals due to presence of protease inhibitor called trypsin inhibitor (TI) in its seeds. The TI accumulates in the soybean seeds primarily as Kunitz trypsin inhibitor (KTI) and to a lower extent as Bowman-Birk trypsin inhibitor (BBTI) [1]. The KTI affects growth and basal metabolism of different animal species upon consuming it. Functionally, the soybean KTI strongly inhibits the enzyme trypsin and thereby reduces food intake by diminishing their digestion and absorption, besides reducing retention of nitrogen absorbed by the consuming animal [2, 3]. Further, it induces pancreatic enzyme’s hyper-secretion and fast stimulation leading to pancreatic hypertrophy and hyperplasia in the animals [4].

Biochemically, KTI is a monomeric and non-glycosylated protein weighing 21.0 kDa, and contains 181 amino acid residues [5]. In soybean, it is encoded by ten independent genes; however, *KTI3* is the most important one, as it predominantly expresses in the seeds [6]. Thirteen iso-forms of KTI3 protein have so far been reported, which are governed by a single gene with multiple alleles [7]. The DNA sequence of the *KTI* recessive allele i.e. *kti* contains one substitution and two deletions, which alters the translation process resulting in reduced levels of KTI in seed embryos [8]. In recent times, detailed aspects of KTI accumulation have been studied with an aim to develop soybean genotypes containing ultra-low or zero KTI [9, 10]. Soybean germplasm line PI542044 maintained at the Indian Institute of Soybean Research, Indore, India found to contain no KTI (KTI free); although it contains the Bowman Birk trypsin inhibitor (BBTI) at a concentration of 14.15 mg g^−1^ seed meal [11]. PI542044, also known as Kunitz soybean, contains the null allele of KTI i.e. *kti* which encodes a truncated protein. This genotype was developed in a backcross program involving Williams 82 and PI157440; hence, it is considered as near iso-genic line of Williams 82 [12]. Usually, for transferring an allele from a donor to an elite recipient genotype, backcrossing is the conventional choice of the breeders; however, the process is tedious and time consuming, particularly for the recessive alleles. Further, introgression of *kti* is complicated by a number of factors viz., (i) *kti* being recessive in inheritance, each conventional backcross generation would be requiring selfing followed by estimation of KTI content in the seeds so as to identify a target plant. Further, a minimum of six backcross generations would be required to recover the recurrent parent genome to a satisfactory level, (ii) the donor (PI542044) being an unadapted germplasm line, it is quite likely to pass some undesirable traits (linkage drag) to the backcross progenies, and (iii) rigorous and seed-destructive biochemical testing will be requiring to estimate the level of trypsin inhibitor in the seeds. Thus, conventional breeding approach offers limited scope for the development of Kunitz trypsin inhibitor free soybean genotypes.

Rapid advances in genomic research and molecular biology led to the development of precise, rapid and efficient molecular markers for speedy development of new cultivars [13]. Marker Assisted Selection (MAS) has been proved to be efficient in introgressing disease and insect resistance genes in several crops [14–19] and the list is expanding with newer successes every year. Recently, three recessive null alleles viz., Kunitz trypsin inhibitor, soybean agglutinin, and P34 allergen nulls were stacked in the background of ‘Williams 82’ and was termed as ‘Triple Null’. The ‘Triple Null’ would have a potential application in conventional feed/food and for immunotherapy to mitigate soybean allergenic responses [20].

Three SSR markers viz., Satt228, Satt409 and Satt429 have been reported to be closely linked (0–10 cm) with the null allele of Kunitz trypsin inhibitor [21]. Effectiveness and utility of these markers has already been proved in different populations [22–25]. In the present investigation, Marker Assisted Backcross Breeding (MABB) approach was employed to introgress the *kti* allele from the donor genotype PI542044 into two elite soybean cultivars viz., DS9712 and DS9814. The KTI free soybean lines developed through this process will meet the long term need of the farmers, consumers and soybean industries.

## Methods

### Plant material

#### Donor genotype

Soybean accession PI542044 which has the null allele of *KTI* was developed from ‘Williams 82’ × PI157440 through 5 backcrossing at the Illinois Agricultural Experimentation Station and USDA-ARS, USA. The seeds of this accession were collected from ICAR-Indian Institute of Soybean Research, Indore, India. PI542044 is an early maturing (85–90 days) genotype with poor agronomic performance under Indian conditions.

#### Recurrent parents

Two popular soybean varieties of North Plain Zone of India viz., DS9712 and DS9814 were chosen as recurrent parents. Both the varieties are high yielding with high degree of resistance to yellow mosaic virus disease (YMD), but contains higher level of KTI (83.37 and 123.96 mg g^−1^seed meal, respectively), and takes longer duration to mature (125–130 days). Therefore, elimination of KTI coupled with reduction of maturity duration would make DS9712 and DS9814 varieties more popular among the end users.

### Target gene and background marker assays

#### Parental polymorphism

Three SSR markers viz., Satt228, Satt409 and Satt429 linked to *kti* were tested for polymorphism between the donor (PI542044) and the two recurrent parents (DS9712 and DS9814). The hybridity testing of the F_1_ plants, and foreground selection in BC_1_ and BC_2_ generations was conducted by these three markers. For assessing the level of recurrent parent genome recovery i.e. background selection, a panel of 93 and 81 polymorphic SSR markers (soybase.org) were used among the DS9712 and DS9814 backcross progenies, respectively.

##### PCR amplification

Genomic DNA was extracted from the young leaves of the selected genotypes following CTAB (cetyltrimethyl ammonium bromide) procedure. Purified DNA was subjected to PCR amplification in 20 μl reaction mixture containing 5.0 μl DNA (20 ng/μl), 2.0 μl 10× buffer, 2.0 μl dNTPs (25 mM), 2.0 μl each forward and reverse SSR primers (30 ng/μl), 0.3 μl *Taq*DNA polymerase (3U/μl) and 6.7 μl double distilled water. Amplification of the template DNAs was performed in thermocycler (Applied Biosystem) as per the following profile: DNA was denatured at 94 °C for 2 min. followed by 35 cycles each consisting of denaturation at 94 °C for 1 min., primer annealing at 49 °C for 2 min., primer elongation at 72 °C for 3 min. Final elongation of the amplicons was allowed to complete at 72 °C for 10 min which was finally put on hold at 4 °C. Amplified products so obtained were resolved on 3 % metaphore agarose gel stained with ethidium bromide and analyzed in Gene Genius Gel Imaging System from Syngene.

### Backcross breeding steps

Due to significant difference in days-to-flowering between the recurrent parents (45 days) and the donor parent (30 days), staggered sowing of the seeds was done in pots to synchronize the flowering for effective hybridization. The hybridization was performed following ‘pollination without emasculation’ technique [26, 27]. During development of the backcross generations, recurrent parent was always used as female parent and respective F_1_ or BC_1_ plants were used as pollen parent. The BC_2_F_1_ plants that tested positive for the target gene (foreground selection) were advanced to BC_2_F_2_ families during January-April, 2012 under controlled condition of National Phytotron Facility, ICAR-Indian Agriculture Research Institute, New Delhi.

### Estimation of RPG content

The marker data was analysed using the software Graphical Genotyping (GGT 2.0). The homozygous recipient allele, homozygous donor allele and heterozygous allele were scored as “A”, “B” and “H” respectively. Double the percentage of markers homozygous for recipient parent (%A) and the percent half of recipient alleles under heterozygous (%H) conditions were summed and averaged over the total number of polymorphic markers to calculate percent recurrent parent genome (RPG %) content.

### Field evaluation

The BC_2_F_2_ families of DS9712 and DS9814 containing the null allele of KTI were raised along with the recurrent parents in the fields of ICAR-Indian Agricultural Research Institute, New Delhi (latitude: 28° 40’N; longitude: 77° 13’ E; altitude: 228 m above mean sea level) during July-October 2013 for assessing the yield and other attributes. Temperature during crop growing period ranged from 25–40 °C with occasional ups and down. Humidity level ranged from 60–80 % with occasional changes caused by monsoon rain. Photoperiod ranged from 10–11 h during sowing to 7–8 h during harvesting period. Each introgressed line was planted in a single row of 3 m length with 45 cm row-to-row and 10 cm plant-to-plant distance. All the recommended agronomic practices were practiced to raise a healthy crop. The observations on days to flowering, plant height (cm), number of pods per plant and seed yield per plant were recorded on each family separately. These parameters were used as additional parameter in identifying introgressed lines resembling the recurrent parents.

### Biochemical analysis

The seeds of the plants homozygous for KTI allele i.e. *ktikti* in BC_2_F_2_ families were biochemically tested through native PAGE for confirming absence of KTI polypeptides. For this purpose, finely ground seed flour (100 mg) was incubated in 1 ml Tris–HCl buffer (pH 8.0) for 30 min. and then centrifuged. Equal volumes of supernatant and 5× sample buffer containing 50 % v/v glycerol, 1.96 % v/v β mercaptoethanol, 0.05 % bromophenol dye and 1 M Tris–HCl (pH 6.8) were loaded on 12 % acrylamide gel in vertical electrophoresis unit (Model SE 600 Ruby®, GE Healthcare) and run at 35 mA for 2 h 30 min. Gels were stained overnight in 0.25 % aqueous solution of coomassie brilliant blue (R-250) in methanol, water and glacial acetic acid (45:45:10) followed by de-staining in methanol, water and glacial acetic acid (45:45:10) solution. Standard trypsin inhibitor protein (21.0 kDa) procured from M/S Sigma Aldrich, Bangalore was loaded in a separate lane for identification of KTI polypeptide in the introgressed lines.

## Results

### Validation of SSR markers linked to *kti*

Three SSR markers viz., Satt228, Satt409 and Satt429 reported to be linked to *kti* were tested for polymorphism between the donor (PI542044) and two recurrent parents viz., DS9712 and DS9814. All the three SSR markers produced polymorphic bands between the donor and the two recurrent parents. Therefore, the markers could be used for checking hybridity of the F_1_ plants as well as in identifying the target plants during foreground selection in the BC_1_ and BC_2_ generations.

### Parental polymorphism survey for background selection markers

For assessing the level of molecular polymorphism between the donor and the recurrent parents, and to select a set of SSR markers for background selection, a total of 290 (between DS9712 and PI542044) (Additional file 1: Table S6) and 180 (between DS9814and PI542044) (Additional file 1: Table S7) markers spanning uniformly across the 20 linkage groups (LG) of soybean were used. In case of DS9712 and PI542044, 93 markers out of 290 (32.06 %) appeared to be polymorphic, while 81 markers out of 180 (43.20 %) were polymorphic between PI542044 and DS9814. Although 17 SSR markers were employed in the carrier chromosome, on average, 5 and 4 markers/chromosome was found to be polymorphic in DS9712 and DS9814 crosses, respectively. The variations in the level of polymorphism indicated level of genetic dissimilarity between the donor and the recurrent parents.

### Development of BC_1_F_1_ and BC_2_F_1_ populations

Using DS9712 and DS9814 as female parents, hybridization was effected pair-wise with the donor parent PI542044. For enhanced success of hybridization, the technique of ‘pollination without emasculation’ [26] was employed, and more than 38 % success of hybridization was obtained. The F_1_ plants confirmed through hybridity test were crossed back to respective recurrent parents and obtained 59 and 52 BC_1_F_1_ seeds from DS9712 and DS9814 crosses, respectively. Similarly, three best BC_1_F_1_ plants from each cross were selected and crossed back to respective recurrent parents to develop 105 and 32 BC_2_F_1_ seeds, respectively. In both the generations, the recurrent parents were used as female parent during hybridization.

### Foreground and background selection in BC_1_F_1_ plants

The foreground selection and background selection was started from BC_1_F_1_ generation. For foreground selection, the DNA samples extracted from the BC_1_F_1_ plants of both the crosses were subjected to PCR amplification with Satt228, Satt409 and Satt429 during July-October 2011. Accordingly, 19 and 10 heterozygous plants were selected from DS9712 and DS9814 crosses, respectively. The selected plants were subsequently subjected to background analysis with polymorphic SSR markers for each cross, separately. The analyses indicated recovery of the recurrent parent genome (RPG) to the tune of 70.43–87.63 % and 60.26–73.78 % with an average recovery of 81.34 % and 64.55 % in DS9712 (Additional file 1: Table S1) and DS9814 (Additional file 1: Table S2) crosses, respectively. Maximum recovery of 87.63 % and 73.78 % was found in the plant No. BC_1_F_1_-7 and BC_1_F_1_-1 of DS9712 and DS9814 crosses, respectively. Two plants viz., BC_1_F_1_-7 and BC_1_F_1_-2 belonging to DS9712 cross found to recover the recurrent parent genome fully at the selected marker loci on 13 chromosomes viz., 3,4, 6, 7, 10, 13, 15 and 4, 5,6, 7, 17 and 19, respectively. The status of RPG recovery in the DS9712 cross-derived plants was higher than that in the DS9814 cross-derived plants. The BC_1_F_1_ plants having higher level of recovery of the recurrent parent genome exhibited higher level of phenotypic similarities with the recurrent parent in respect of growth habit and pubescence color. Based on extent of RPG recovery, 3 plants from DS9712 cross and 2 plants from DS9814 cross were selected and backcrossed with the recurrent parent to produce the BC_2_F_1_ seeds. The selected plants viz., BC_1_F_1_-7, BC_1_F_1_-2, BC_1_F_1_-35 from DS9712 cross and BC_1_F_1_-1 and BC_1_F_1_-5 from DS9814 cross had RPG recovery of 87.63, 87.09, 87.07, and 73.78, 71.16 %, respectively.

### Foreground and background selection in BC_2_F_1_ plants

A total of 105 and 32 BC_2_F_1_ seeds were harvested from the selected plants of DS9712 and DS9814 crosses, respectively. The seeds were grown in the National Phytotron Facility (NPF), IARI, New Delhi during January-April 2012, of which 73 plants of DS9712 cross and 29 plants of the DS9814 cross survived. As like in the BC_1_F_1_ generation, the genomic DNA extracted from all the BC_2_F_1_ plants was subjected to foreground selection. Accordingly, 38 and 15 plants, respectively from the DS9712 and DS9814 crosses were selected for background selection. The background markers which exhibited heterozygous genotype in the BC_1_F_1_ plants were only used for background analysis in this generation. The recovery of the RPG in the selected plants of both crosses ranged from 83.30 to 98.82 % (Additional file 1: Table S3). Such level of RPG recovery is much higher than expected (87.25 %) through conventional backcrossing approach. A group of 13 plants found to have more than 96 % recovery of the RPG. The highest recovery was found in plant No. DI-2 (98.92 %) followed by AI-9 (98.32 %), AI-2 (97.84 %) and DI-1 (97.84 %). In the plant DI-2, only two marker loci were found to be heterozygous, where as in plant No. AI-9, AI-2 and DI-3, three to four markers loci remained in heterozygous conditions; rest of the background selection makers had attained homozygosity indicating full recovery of the recurrent parent genome. The AI and DI plants denote introgressed lines derived from the DS9712 cross. In the backcross-derived plants of DS9814 cross, the recovery of recurrent parent genome ranged from 83.3 to 91.67 % (Table 1). In 7 out of fifteen selected plants, the recovery of RPG was more than 90 %, which is again far higher than expected theoretical average recovery percentage i.e. 87.50 %.

**Table 1:** Summary of the foreground and background selections carried out during backcross generations

Generation	Selection	Cross combination	No. of plants screened	No. of heterozygous plants	No. of plants tested for background selection	No. of background markers surveyed	RPG content range (%)
F_1_	Foreground selection	DS9712 x PI542044	82	78	-	-	-
F_1_	Foreground selection	DS9814 x PI542044	69	53	-	-	-
BC_1_F_1_	Foreground & background selection	DS9712 x PI542044	59	19	19	93	70.43–87.63
BC_1_F_1_	Foreground & background selection	DS9814 x PI542044	52	10	10	81	60.26–73.78
BC_2_F_1_	Foreground & backgroundselection	DS9712 x PI542044	73	38	38	23–24	93.01–98.82
BC_2_F_1_	Foreground & background selection	DS9814 x PI542044	29	15	15	41–45	83.3–91.67

**Fig. 1:**
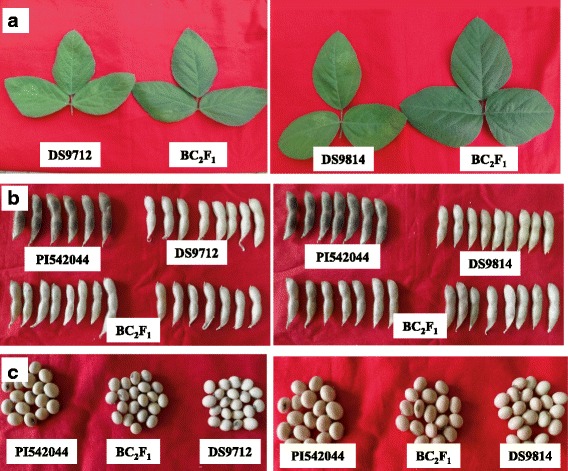
Phenotypic comparison of the leaf (**a**), pods (**b**) and seeds (**c**) between recurrent and MABB derived plants in BC_2_F_1_ generation

The BC_2_F_1_ plants from DS9712 and DS9814 crosses were compared with respective recurrent parents for phenotypic qualitative traits like pubescence color, pod color, seed shape, etc., which indicated higher level of similarity of the BC_2_ - derived plants with their respective recurrent parent. In case of DS9814-derived plants, the plants looked nearly same as the recurrent parent (Fig. 1).

### Recombinant selection

**Fig. 2:**
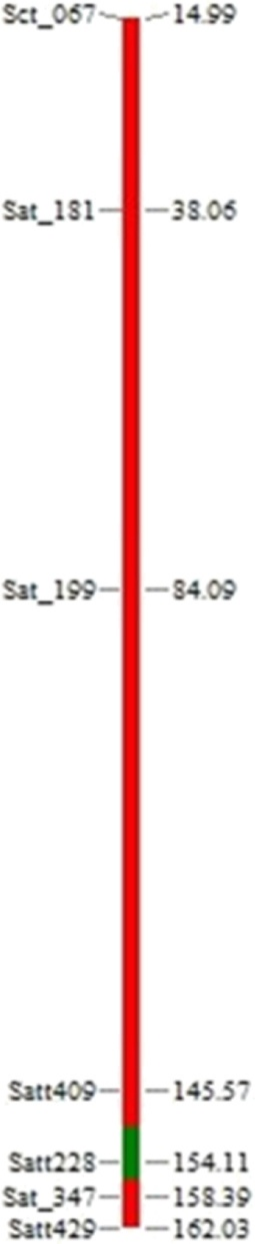
Graphical genotype of a double recombinant plant (AI-2) that has completely recovered the recurrent parent allele (red color) except at locus Satt228 during BC_2_F_1_ generation

Recombinant selection was carried out with the help of two markers viz., Satt409 and Satt429 located on either side of the allele of interest (*kti*). In BC_1_F_1_ generation no double or single recombinants were found as flanking markers Satt409 and Satt429 appeared as heterozygous. However, in BC_2_ generation, 9 plants with single recombination and one plant (AI-2) with double recombination were recovered among the DS9712-derived plants (Additional file 1: Table S4). Similarly, in the DS9814-derived plants, four single recombinants recovered. The graphical representation of the double recombinant (AI-2) is presented in Fig. 2.

### Field evaluation of BC_2_F_2_ plants

**Fig. 3:**
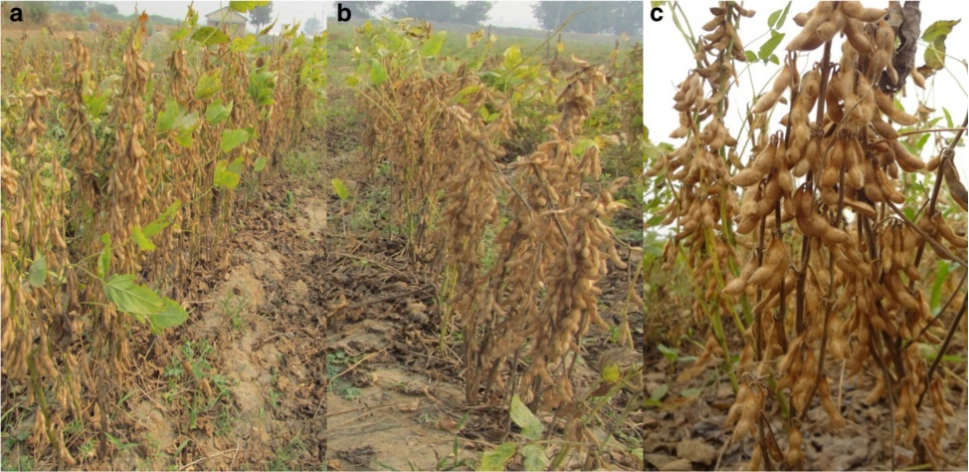
Evaluation of introgressed BC_2_F_2_ families under field condition. **a**: Recurrent parent (DS9712), (**b**) and (**c**): KTI free BC_2_F_2_ families

The BC_2_F_2_ families introgressed with *kti* allele were evaluated during 2012 (July-October) in the experimental field of IARI, New Delhi-12 (Fig. 3). The standard package of practices was followed to raise a good crop. Observations were recorded from each individual of the families on morpho- phenotypic traits. Nearly all the families flowered one week earlier (37 days) than the recurrent parents in both the crosses (Additional file 1: Table S5). The KTI free BC_2_F_2_ plants which were confirmed by native PAGE were found to yield higher than the recurrent parents (Table 2).

**Table 2:** Agronomic performance of the six BC_2_F_2_plants under field condition

BC_2_F_2_Plant No.	Days to flowering	Plant height (cm)	No. of pods	Seed yield/plant (gm)
*AI-1-1	38	36.50	146	34.50
AI-2-3	37	35.00	198	38.00
AI-11-2	36	38.50	150	30.00
AI-51-2	39	28.00	159	36.50
*DI-3-12	36	40.50	126	36.25
DI-3-7	39	36.00	119	30.00
DS9712 (Recurrent parent)	45	39.00	110	22.00
DS9814 Recurrent parent)	46	38.00	115	22.50
PI542044 (Donor parent)	30	32.00	45	12.00

*AI and DI denotes introgressed lines of DS9712 cross

### Biochemical confirmation

**Fig. 4:**
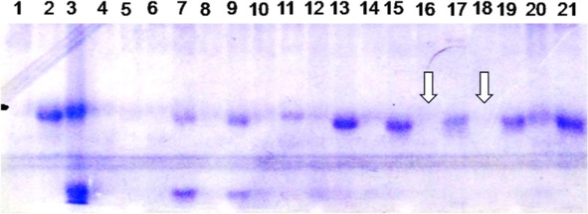
Biochemical confirmation of absence of KTI peptides in the selected plants; Lane 1: Donor parent (PI542044), Lane 2 and 21: KTI standard (M), Lane 3: Recurrent parent (DS9712); Lane 4–20: Segregating plants; Lane 16 and 18: KTI free plants

The plants homozygous for *kti* allele i.e. *ktikti* were identified in the BC_2_F_2_ generation by linked SSR markers. Seeds of such selected plants were tested for presence/absence of KTI peptides through native polyacrylamide gel electrophoresis (PAGE). In total, 6 plants were found to be free from KTI peptides (Fig. 4). It thus confirmed successful transfer of the *kti* allele into two popular varieties of soybean through marker assisted backcross breeding approach. The *kti* allele is unable to synthesize functional KTI peptide and hence the plants with such allele in homogyzous state were free from KTI peptide.

## Discussion

One of the major constraints limiting wider acceptance of soy food and feed is the presence of Kunitz trypsin inhibitor (21.0 kDa) in its seeds [28]. Usually, heat treatment is given to the soy-flour to eliminate the KTI. However, the thermal treatment is not fully effective in elimination of the KTI peptides [29]. At house-hold level, soybean seeds are boiled before grinding and mixing with wheat flour (@1:9) for making quality *chapatti*. All these steps increase cost of production besides affecting solubility and quality of seed proteins. Presently in India, none of the soybean varieties are free from KTI [23]. In this study, null allele of KTI from PI542044 was introgressed into two popular soybean varieties viz., DS9712 and DS9814 through marker assisted backcross breeding (MABB) approach so as to develop improved soybean lines free from KTI. The approach of foreground selection used here facilitated easy identification of the targeted plants at seedling stage and thus helped in reducing the population size by half in a backcross generation. Similarly, the background selection helped in reducing the time required for product development [30–32]. Further, MABB approach helped in efficient tracking of the recessive allele (*kti*) under consideration. In the absence of linked molecular marker(s), it would not have been possible to identify a KTI free plant without analyzing the seeds biochemically. However, in the early generations, seed is a critical factor and difficult to spare for destructive biochemical analysis. Thus, molecular breeding approach added speed and accuracy to the selection of KTI free plants in various generations.

Number of backcross generation to be employed in MABB is an important issue; some feels that two backcross generations are sufficient while others advocate for three. In fact, it depends on the genomic constitution of the plants selected for backcrossing in one hand, and nature of the donor parent genome, on the other. If background selection is done rigorously and plants with higher RPG recovery are selected as pollen source, two backcross generations would be sufficient. However, wherever background selection is not done in first few generations, three backcross generations is the answer. In this experiment, plants with 98.92 % recovery of the RPG were recovered in BC_2_F_1_ generation as against 87.25 % recovery expected theoretically. This advancement is the result of conscious selection of pollen-source plants that had highest recovery (87 %) of the RPG in BC_1_F_1_generation. The range of recovery of the RPG in BC_2_F_1_ generation ranged from 83.3 to 98.92 %. Such rapid recovery of the recurrent parent genome would reduce the time requirement in introgression of gene(s) through molecular breeding approach. Similar success of molecular breeding has been reported in some other crops as well [33–39]. Recently, eight quantitative trait loci (QTLs)/genes were pyramided for four grain quality traits (high grain weight, high grain protein content, pre-harvest sprouting tolerance, and desirable high-molecular-weight glutenin subunits and resistance against three rust diseases) in bread wheat by MABB approach [18]. Marker-assisted backcrossing was employed to introgress resistance to *Fusarium* wilt Race 1 and *Ascochyta blight* in Chickpea [19]. Similarly in maize, using marker-assisted backcrossing a major QTL for oil content was transferred to an elite hybrid [40]. In soybean, marker-assisted backcrossing was used to transfer a null allele of α-subunit of soybean β-conglycin into Chinese cultivar for developing improved lines devoid of β-conglycinin [41].

Molecular marker-based selection supplemented with phenotypic selection may be considered as the best option for identification of target plants in the shortest possible time. It helps not only in identification of plants with the desirable traits but also ensures selective elimination of the undesirable traits from the selected plants. In this experiment, the selected plants were not only free from KTI but also similar to the recurrent parents in phenotypic expressions. The selected plants were also resistant to yellow mosaic virus disease. Similarly, the selected plants found to mature 5–7days earlier to the recurrent parents. It would make the resultant genotypes fit in soybean-wheat cropping sequence in the soybean growing belt of India.

It is often said that trypsin inhibitor in plants plays some defensive role in protecting the plants from insect pests and diseases [42, 43]. Therefore, its elimination might make the plants vulnerable to such pests and diseases. It is however, reported that the BBI fraction of the trypsin inhibitor offers the required resistances to the KTI-free plants. The BBI fraction is also reported to have some protective role in human health [44, 45].

The problem of linkage drag is a common feature in backcross breeding program involving un-adapted germplasm. In this study, to avoid the problem of linkage drag, recombinant selection approach was carried out by employing two flanking markers. Accordingly, plants having shortest introgressed segment of donor chromosome around the target loci were selected in BC_2_ generation. As a result, one double recombinant and fourteen single recombinant plants could be recovered. The double recombinant plant (AI-2) had least size of the introgressed segments on carrier chromosome. Recovery of such recombinants at the target locus is not easily possible in conventional backcross breeding programme.

The resultant plants of MABB should not only have the desired trait but also to match the recurrent parent in performance in yield and other phenotypic traits. In this experiment, the homozygous plants selected in progeny rows were compared for their phenotypic performances including per plant yield. A total of 38 and 15 BC_2_F_2_ families respectively from DS9712 and DS9814 populations were grown under field conditions to evaluate for their agronomic performance. The results indicated that four families along with six KTI free plants recorded higher yield than recurrent parent. Moreover, they were about one week early in maturity as compared to the respective recurrent parents. This would make the genotypes suitable for growing in rice-wheat cropping sequence easily.

## Conclusions

In the present investigation, MABB approach was employed for speedy introgression of the null allele of KTI into two high yielding soybean cultivars viz., DS9712 and DS9814. Unlike conventional backcross breeding approach, the MABB helped in quick recovery of the recurrent parent genome. The six KTI-free lines developed in this study would have direct industrial application in manufacturing primary soy products like tofu, soy milk, soy-nuts, etc., without pre-thermal treatment to the soy-flour. Also, raw soybean grains of these KTI-free breeding lines would be fit to supplement directly in the feed of non-ruminants. The trypsin inhibitor free lines so developed will open up vistas for development of trypsin inhibitor free varieties for large scale use in soybean industries.

## Abbreviations

IARI, Indian agricultural research institute; KTI, Kunitz trypsin inhibitor; MABB, Marker assisted backcross breeding; PAGE, Polyacrylamide gel electrophoresis; RPG, Recurrent parent genome; TI, Trypsin inhibitor

## Additional file

Additional file 1: Table S1. Recovery of recurrent parent (DS9712) genome in BC_1_F_1_ generation. **Table S2.** Recovery of recurrent parent (DS9814) genome in BC_1_F_1_ generation. **Table S3.** Recovery of recurrent parent genome (RPG) in BC_2_F_1_ generation. **Table S4.** Recombinant selections in BC_2_F_1_ generation of DS9712 cross. **Table S5.** Mean performance of the BC_2_F_2_ families under field conditions. **Table S6.** Details of the SSR markers screened for parental polymorphism between the recurrent (DS9712) and donor parent (PI542044). **Table S7.** Details of the Simple Sequence Repeat markers screened for parental polymorphism between the recurrent (DS9814) and donor parent (PI542044). (DOC 605 kb)
