# Supplementary material for: Introgression of null allele of Kunitz trypsin inhibitor through marker-assisted backcross breeding in soybean (Glycine max L. Merr.)
Source: BMC Genet. 2016 Jul 12;17:106. doi: 10.1186/s12863-016-0413-2 (PMC4943013; doi:10.1186/s12863-016-0413-2)
Supplement: Additional file 1: Table S1. — Recovery of recurrent parent (DS9712) genome in BC1F1 generation. Table S2. Recovery of recurrent parent (DS9814) genome in BC1F1 generation. Table S3. Recovery of recurrent parent genome (RPG) in BC2F1 generation. Table S4. Recombinant selections in BC2F1 generation of DS9712 cross. Table S5. Mean performance of the BC2F2 families under field conditions. Table S6. Details of the SSR markers screened for parental polymorphism between the recurrent (DS9712) and donor parent (PI542044). Table S7. Details of the Simple Sequence Repeat markers screened for parental polymorphism between the recurrent (DS9814) and donor parent (PI542044). (DOC 605 kb) [file 12863_2016_413_MOESM1_ESM.doc]

**Table S1 Recovery of recurrent parent (DS9712) genome in BC_1_F_1_** generation

|  | Plant No. | | | | | | | | | | | | | | | | | | |
| --- | --- | --- | --- | --- | --- | --- | --- | --- | --- | --- | --- | --- | --- | --- | --- | --- | --- | --- | --- |
|  | 2 | 4 | 5 | 7 | 9 | 12 | 13 | 14 | 17 | 21 | 23 | 28 | 31 | 33 | 35 | 38 | 40 | 41 | 42 |
| **A (%)** | 74.19 | 68.81 | 67.74 | 75.26 | 73.11 | 72.64 | 69.89 | 59.14 | 67.74 | 60.21 | 70.96 | 56.98 | 72.64 | 48.38 | 74.19 | 40.86 | 49.46 | 46.23 | 44.08 |
| **H (%)** | 25.80 | 31.18 | 32.25 | 24.73 | 26.88 | 27.95 | 30.10 | 40.86 | 32.25 | 39.78 | 29.03 | 43.01 | 27.95 | 51.61 | 25.80 | 59.14 | 50.53 | 53.76 | 55.91 |
| **RPG (%)** | 87.09 | 84.40 | 83.87 | 87.63 | 86.55 | 86.02 | 84.94 | 79.57 | 83.87 | 80.10 | 85.48 | 78.49 | 86.02 | 74.19 | 87.07 | 70.43 | 74.73 | 73.11 | 72.02 |

**A: Homozygous recurrent parent allele; H: Heterozygous allele; RPG: Recovery of recurrent parent genome**

**Table S2 Recovery of recurrent parent (DS9814) genome in BC_1_F_1_** generation

|  | Plant No. | | | | | | | | | |
| --- | --- | --- | --- | --- | --- | --- | --- | --- | --- | --- |
|  | 1 | 5 | 8 | 11 | 24 | 27 | 35 | 38 | 46 | 48 |
| **A (%)** | 47.34 | 42.30 | 29.49 | 29.49 | 26.92 | 26.92 | 23.08 | 21.34 | 24.36 | 21.79 |
| **H (%)** | 52.56 | 57.69 | 70.51 | 70.51 | 73.08 | 73.08 | 76.92 | 78.21 | 75.64 | 78.21 |
| **RPG (%)** | 73.78 | 71.16 | 64.74 | 64.74 | 63.46 | 63.46 | 61.54 | 60.26 | 62.18 | 60.26 |

**A: Homozygous recurrent parent allele; H: Heterozygous allele; RPG: Recovery of recurrent parent genome**

**Table S 3. Recovery of recurrent parent genome (RPG) in BC_2_F_1_ generation.**

| **BC_2_F_1_ plant no.#** | **A (%)** | **H (%)** | **RPG (%)** |
| --- | --- | --- | --- |
| AI-2 | 95.69 | 4.30 | 97.84 |
| AI-4 | 92.47 | 7.50 | 96.23 |
| AI-5 | 86.02 | 13.97 | 93.01 |
| AI-9 | 96.77 | 3.22 | 98.38 |
| AI-10 | 91.39 | 8.60 | 95.69 |
| AI-14 | 88.17 | 11.82 | 94.08 |
| AI-17 | 87.09 | 12.90 | 93.54 |
| AI-29 | 94.62 | 5.37 | 97.31 |
| AI-32 | 84.90 | 15.05 | 92.47 |
| AI-42 | 90.32 | 9.67 | 95.16 |
| AI-44 | 89.24 | 10.75 | 94.26 |
| AI-61 | 93.54 | 6.45 | 96.77 |
| BI-1 | 86.02 | 13.97 | 93.01 |
| BI-3 | 88.17 | 11.82 | 94.08 |
| DI-1 | 95.69 | 4.30 | 97.84 |
| DI-2 | 97.84 | 2.15 | 98.92 |
| DI-3 | 92.42 | 7.52 | 96.23 |
| KII-1 | 76.92 | 23.08 | 88.46 |
| KII-2 | 78.21 | 21.79 | 89.10 |
| KII-3 | 83.33 | 16.67 | 91.67 |
| FII-1 | 74.36 | 25.64 | 87.18 |
| FII-2 | 80.77 | 19.23 | 90.38 |
| EII-1 | 79.49 | 20.51 | 89.74 |
| EII-2 | 82.05 | 17.95 | 91.03 |
| HII-1 | 80.77 | 19.23 | 90.38 |
| HII-2 | 83.33 | 16.67 | 91.67 |
| HII-3 | 83.33 | 16.67 | 91.67 |
| HII-4 | 80.77 | 19.23 | 90.38 |
| I(II)-1 | 76.92 | 23.08 | 88.46 |
| I(II)-2 | 71.79 | 28.21 | 85.89 |
| I(II)-4 | 66.67 | 33.33 | 83.33 |
| J(II)-1 | 74.36 | 25.64 | 87.18 |

A=Homozygous segments, H=Heterozygous segments

#AI –DI plants are progeny of DS9712 and rests are from DS9814 crosses

**Table S4 Recombinant selection in BC_2_F_1_** generation of DS9712 cross

| **Plant No.** | **Satt228** | **Satt409** | **Satt429** | **Plant No.** | **Satt228** | **Satt409** | **Satt429** |
| --- | --- | --- | --- | --- | --- | --- | --- |
| AI-1 | 2* | 2 | 2 | AI-38 | 3 | 3 | 3 |
| AI-2 | 2 | 3 | 3 | AI-39 | 3 | 3 | 3 |
| AI-3 | 3 | 3 | 2 | AI-40 | 2 | 2 | 3 |
| AI-4 | 2 | 2 | 2 | AI-41 | 2 | 2 | 3 |
| AI-5 | 2 | 2 | 2 | AI-42 | 2 | 2 | 2 |
| AI-6 | 3 | 3 | 3 | AI-43 | 2 | 2 | 2 |
| AI-7 | 2 | 2 | 3 | AI-44 | 2 | 2 | 2 |
| AI-8 | 3 | 3 | 3 | AI-45 | 3 | 2 | 2 |
| AI-9 | 2 | 2 | 3 | AI-46 | 2 | 2 | 3 |
| AI-10 | 2 | 2 | 2 | AI-47 | 3 | 3 | 3 |
| AI-11 | 2 | 2 | 2 | AI-48 | 2 | 2 | 2 |
| AI-12 | 3 | 3 | 3 | AI-49 | 2 | 2 | 2 |
| AI-13 | 3 | 3 | 3 | AI-50 | 2 | 2 | 2 |
| AI-14 | 2 | 2 | 2 | AI-51 | 2 | 2 | 2 |
| AI-15 | 3 | 3 | 2 | AI-52 | 3 | 3 | 3 |
| AI-16 | 3 | 3 | 3 | AI-53 | 2 | 2 | 2 |
| AI-17 | 2 | 2 | 3 | AI-54 | 3 | 3 | 3 |
| AI-18 | 3 | 3 | 3 | AI-55 | 3 | 3 | 3 |
| AI-19 | 3 | 3 | 3 | AI-56 | 2 | 2 | 3 |
| AI-20 | 3 | 3 | 3 | AI-57 | 3 | 3 | 3 |
| AI-21 | 3 | 3 | 3 | AI-58 | 2 | 2 | 3 |
| AI-22 | 3 | 3 | 3 | AI-59 | 3 | 3 | 3 |
| AI-23 | 3 | 3 | 3 | AI-60 | 3 | 3 | 3 |
| AI-24 | 3 | 3 | 3 | AI-61 | 2 | 2 | 2 |
| AI-25 | 3 | 3 | 3 | AI-62 | 2 | 2 | 2 |
| AI-26 | 3 | 2 | 2 | AI-63 | 2 | 2 | 2 |
| AI-27 | 3 | 3 | 3 | AI-64 | 2 | 2 | 2 |
| AI-28 | 2 | 2 | 2 | AI-65 | 3 | 3 | 3 |
| AI-29 | 2 | 2 | 2 | AI-66 | 3 | 3 | 3 |
| AI-30 | 3 | 3 | 3 | AI-67 | 3 | 3 | 3 |
| AI-31 | 2 | 2 | 2 | BI-1 | 2 | 2 | 3 |
| AI-32 | 2 | 2 | 3 | BI-2 | 3 | 3 | 3 |
| AI-33 | 3 | 3 | 3 | BI-3 | 2 | 2 | 2 |
| AI-34 | 2 | 2 | 2 | DI-1 | 2 | 2 | 3 |
| AI-35 | 3 | 2 | 2 | DI-2 | 2 | 2 | 3 |
| AI-36 | 2 | 2 | 2 | DI-3 | 2 | 2 | 2 |
| AI-37 | 3 | 3 | 3 |  |  |  |  |

* 2= Heterozygous allele, 3= Homozygous allele

**Table S5 Mean performance of the BC_2_F_2_ families under field conditions**

| **BC_2_F_2_ Families** | **Days to flower** | **Plant height (cm)** | **No. of pods** | **Seed yield/plant (g)** |
| --- | --- | --- | --- | --- |
| AI-1 | 38.67 | 25.33 | 91.33 | 20.67 |
| AI-2 | 40.17 | 25.42 | 105.33 | 19.50 |
| AI-4 | 41.40 | 25.60 | 116.60 | 23.60 |
| AI-5 | 39.50 | 18.90 | 60.25 | 14.40 |
| AI-7 | 48.00 | 17.25 | 63.50 | 17.25 |
| AI-9 | 42.83 | 25.50 | 94.00 | 19.67 |
| AI-10 | 47.66 | 19.67 | 41.33 | 11.66 |
| AI-11 | 37.83 | 28.67 | 80.33 | 20.83 |
| AI-14 | 43.33 | 17.00 | 51.67 | 12.67 |
| AI-17 | 41.25 | 25.25 | 100.50 | 22.75 |
| AI-28 | 41.40 | 30.90 | 118.50 | 22.10 |
| AI-29 | 42.00 | 24.67 | 83.22 | 19.89 |
| AI-31 | 44.00 | 30.29 | 101.57 | 23.71 |
| AI-32 | 38.00 | 24.00 | 144.00 | 29.00 |
| AI-34 | 39.57 | 21.71 | 92.86 | 22.29 |
| AI-36 | 44.46 | 20.04 | 60.31 | 12.77 |
| AI-40 | 40.00 | 26.33 | 96.11 | 21.00 |
| AI-41 | 40.00 | 26.33 | 96.11 | 21.00 |
| AI-42 | 39.00 | 25.83 | 107.83 | 25.00 |
| AI-43 | 40.55 | 23.33 | 72.56 | 16.67 |
| AI-44 | 39.66 | 22.00 | 86.22 | 19.78 |
| AI-46 | 42.33 | 21.67 | 68.67 | 14.00 |
| AI-48 | 39.00 | 21.67 | 62.00 | 16.00 |
| AI-49 | 40.50 | 18.00 | 91.00 | 19.50 |
| AI-50 | 38.66 | 22.83 | 72.33 | 18.00 |
| AI-51 | 38.00 | 24.50 | 100.50 | 22.75 |
| AI-53 | 40.50 | 18.65 | 56.25 | 13.00 |
| AI-56 | 43.71 | 24.57 | 73.29 | 15.86 |
| AI-58 | 42.00 | 24.00 | 87.00 | 19.00 |
| AI-61 | 41.50 | 16.00 | 34.50 | 9.50 |
| AI-62 | 38.66 | 24.33 | 87.67 | 22.33 |
| AI-63 | 41.60 | 20.80 | 60.00 | 13.00 |
| AI-64 | 46.50 | 19.25 | 53.75 | 11.00 |
| BI-1 | 38.50 | 19.25 | 14.00 | 4.00 |
| BI-3 | 37.75 | 20.63 | 94.25 | 22.25 |
| DI-1 | 39.00 | 30.00 | 108.00 | 22.00 |
| DI-2 | 38.83 | 23.83 | 83.50 | 20.00 |
| DI-3 | 37.63 | 36.18 | 105.27 | 25.18 |

**Table S6 Details of the SSR markers screened for parental polymorphism between the recurrent (DS9712) and donor parent (PI542044)**

| **Sl.No.** | **Chrom.** | **SSR marker** | **Distance(cM)** | **P/M** |  | **Sl.No.** | **Chrom.** | **SSR marker** | **Distance(cM)** | **P/M** |
| --- | --- | --- | --- | --- | --- | --- | --- | --- | --- | --- |
| 1 | 5 | Satt175 | 66.99 | P |  | 44 | 11 | Satt444 | 85.92 | M |
| 2 | 5 | Satt511 | 94.20 | P |  | 45 | 11 | Satt583 | 84.19 | P |
| 3 | 5 | Sat_356 | 42.80 | M |  | 46 | 11 | Satt484 | 118.53 | M |
| 4 | 5 | Satt599 | 85.58 | M |  | 47 | 11 | Sat_364 | 84.25 | M |
| 5 | 5 | Satt175 | 66.99 | M |  | 48 | 11 | Sct_026 | 78.13 | M |
| 6 | 5 | Sat_267 | 78.45 | P |  | 49 | 11 | Sat_411 | 30.87 | M |
| 7 | 5 | Satt545 | 71.39 | P |  | 50 | 11 | Satt322 | 80.89 | P |
| 8 | 5 | Sat174 | 88.58 | P |  | 51 | 11 | Satt415 | 82.89 | M |
| 9 | 5 | Satt591 | 31.14 | M |  | 52 | 11 | Sat_156 | 35.00 | P |
| 10 | 5 | Satt385 | 64.74 | P |  | 53 | 11 | Sat_149 | 54.09 | P |
| 11 | 5 | Sat_271 | 97.76 | M |  | 54 | 11 | Satt251 | 36.48 | M |
| 12 | 5 | Satt200 | 92.89 | P |  | 55 | 11 | Satt197 | 46.39 | M |
| 13 | 5 | Sat_368 | 14.37 | M |  | 56 | 11 | Satt453 | 123.96 | M |
| 14 | 5 | Satt236 | 93.23 | M |  | 57 | 14 | Satt560 | 97.12 | M |
| 15 | 5 | Satt717 | 51.95 | P |  | 58 | 14 | Satt556 | 73.21 | M |
| 16 | 5 | Satt619 | 69.21 | P |  | 59 | 14 | Sat_287 | 31.88 | M |
| 17 | 5 | Satt050 | 46.45 | M |  | 60 | 14 | Satt556 | 73.21 | P |
| 18 | 5 | Satt211 | 95.96 | M |  | 61 | 14 | Sct_034 | 51.45 | M |
| 19 | 5 | Satt648 | 59.18 | M |  | 62 | 14 | Satt168 | 55.2 | P |
| 20 | 5 | Satt593 | 25.56 | M |  | 63 | 14 | Sat_355 | 66.24 | M |
| 21 | 8 | Satt632 | 51.51 | M |  | 64 | 14 | Satt467 | 17.77 | M |
| 22 | 8 | Satt377 | 90.84 | M |  | 65 | 14 | Sat_230 | 72.08 | M |
| 23 | 8 | Satt378 | 165.73 | M |  | 66 | 14 | Sat_177 | 7.84 | M |
| 24 | 8 | Sat538 | 159.63 | M |  | 67 | 14 | Satt601 | 67.73 | M |
| 25 | 8 | BE820148 | 35.93 | M |  | 68 | 14 | Satt534 | 87.59 | M |
| 26 | 8 | Satt538 | 159.63 | M |  | 69 | 14 | Sat_287 | 31.88 | P |
| 27 | 8 | Sat_347 | 158.39 | P |  | 70 | 14 | Satt534 | 87.59 | M |
| 28 | 8 | GMGNOD2B | 58.44 | M |  | 71 | 14 | Satt070 | 72.89 | P |
| 29 | 8 | Satt538 | 159.63 | M |  | 72 | 14 | Satt687 | 113.61 | M |
| 30 | 8 | Sat_181 | 38.06 | P |  | 73 | 4 | Sct_186 | 9.02 | M |
| 31 | 8 | Satt707 | 116.62 | M |  | 74 | 4 | Satt690 | 5.36 | M |
| 32 | 8 | Sat_215 | 53.75 | M |  | 75 | 4 | Sat_337 | 32.1 | M |
| 33 | 8 | Sat_199 | 84.09 | P |  | 76 | 4 | Satt338 | 123.79 | P |
| 34 | 8 | Sat_377 | 116.64 | M |  | 77 | 4 | Satt139 | 74.46 | P |
| 35 | 8 | Sct_067 | 14.99 | P |  | 78 | 4 | Satt565 | 0 | M |
| 36 | 8 | Satt177 | 36.77 | M |  | 79 | 4 | Satt194 | 26.35 | M |
| 37 | 8 | Satt_069 | 108.70 | M |  | 80 | 4 | Sat_077 | 70 | M |
| 38 | 11 | Satt665 | 96.36 | M |  | 81 | 4 | Satt180 | 127.77 | M |
| 39 | 11 | Sat_272 | 14.32 | M |  | 82 | 4 | Satt161 | 73.29 | M |
| 40 | 11 | Sat_261 | 32.95 | P |  | 83 | 4 | Sat_207 | 87.31 | M |
| 41 | 11 | Sat_270 | 21.99 | M |  | 84 | 4 | Sat_367 | 28.04 | P |
| 42 | 11 | Sat_411 | 30.87 | M |  | 85 | 4 | AW277661 | 74.79 | M |
| 43 | 11 | Sat_095 | 81.31 | P |  | 86 | 4 | Satt361 | 75.52 | M |

Contd....

| **Sl.No** | **Chrom** | | **SSR marker** | | **Distance(cM)** | | **P/M** |  | **Sl.No** | **Chrom** | | **SSR marker** | **Distance(cM)** | | **P/M** |
| --- | --- | --- | --- | --- | --- | --- | --- | --- | --- | --- | --- | --- | --- | --- | --- |
| 87 | 4 | Satt396 | | 24.11 | | P | |  | 131 | 2 | Sat_289 | | 131.92 | M | |
| 88 | 4 | Satt164 | | 132.46 | | M | |  | 132 | 2 | Sat142 | | 86.49 | M | |
| 89 | 6 | Satt460 | | 117.77 | | M | |  | 133 | 2 | Satt296 | | 52.61 | M | |
| 90 | 6 | Satt520 | | 42.37 | | M | |  | 134 | 2 | Satt542 | | 53.02 | P | |
| 91 | 6 | AW734043 | | 4.22 | | P | |  | 135 | 2 | Satt558 | | 43.91 | M | |
| 92 | 6 | Satt100 | | 113.96 | | M | |  | 136 | 2 | Sat_211 | | 38.44 | M | |
| 93 | 6 | Satt640 | | 30.47 | | M | |  | 137 | 17 | GMHSP179 | | 99.04 | M | |
| 94 | 6 | Satt227 | | 26.65 | | M | |  | 138 | 17 | Sat_220 | | 128.73 | P | |
| 95 | 6 | Satt643 | | 94.65 | | P | |  | 139 | 17 | Satt311 | | 84.62 | P | |
| 96 | 6 | Sat_366 | | 51.84 | | M | |  | 140 | 17 | Satt186 | | 105.45 | M | |
| 97 | 6 | Sat_252 | | 127.00 | | M | |  | 141 | 17 | Sat_209 | | 85.64 | M | |
| 98 | 6 | Satt202 | | 126.24 | | P | |  | 142 | 17 | Satt662 | | 87.88 | P | |
| 99 | 6 | Sat_238 | | 117.46 | | P | |  | 143 | 17 | Satt082 | | 87.25 | M | |
| 100 | 6 | Satt286 | | 101.75 | | M | |  | 144 | 17 | Sat_365 | | 87.35 | P | |
| 101 | 6 | Satt170 | | 70.56 | | M | |  | 145 | 17 | Satt277 | | 107.59 | M | |
| 102 | 1 | Satt408 | | 106.69 | | M | |  | 146 | 17 | Sat_001 | | 92.12 | M | |
| 103 | 1 | Satt342 | | 48.14 | | M | |  | 147 | 17 | Satt528 | | 86.34 | P | |
| 104 | 1 | Satt368 | | 43.84 | | M | |  | 148 | 15 | Satt685 | | 56.70 | P | |
| 105 | 1 | Satt383 | | 56.57 | | M | |  | 149 | 15 | Satt230 | | 71.31 | M | |
| 106 | 1 | Satt267 | | 57.34 | | M | |  | 150 | 15 | Satt483 | | 44.98 | M | |
| 107 | 1 | Sat_110 | | 62.52 | | M | |  | 151 | 15 | Sat_380 | | 43.29 | M | |
| 108 | 1 | Satt352 | | 48.14 | | M | |  | 152 | 15 | Sat_376 | | 69.23 | M | |
| 109 | 1 | Satt147 | | 108.89 | | P | |  | 153 | 15 | Satt213 | | 3.72 | M | |
| 110 | 1 | Satt370 | | 60.99 | | M | |  | 154 | 15 | Satt691 | | 19.7 | P | |
| 111 | 1 | Satt482 | | 45.75 | | P | |  | 155 | 15 | Satt720 | | 20.8 | P | |
| 112 | 1 | Sat_413 | | 5.93 | | P | |  | 156 | 15 | Satt231 | | 70.23 | M | |
| 113 | 1 | Satt320 | | 46.80 | | P | |  | 157 | 15 | Sat_112 | | 8.67 | M | |
| 114 | 1 | AW781285 | | 67.78 | | M | |  | 158 | 15 | Satt381 | | 44.99 | M | |
| 115 | 1 | Satt532 | | 49.07 | | M | |  | 159 | 15 | Satt212 | | 32.27 | P | |
| 116 | 1 | Satt531 | | 40.87 | | M | |  | 160 | 13 | Satt656 | | 135.12 | M | |
| 117 | 1 | Satt407 | | 99.59 | | M | |  | 161 | 13 | Satt252 | | 16.08 | M | |
| 118 | 1 | Sat_036 | | 75.25 | | P | |  | 162 | 13 | Sct_188 | | 85.33 | P | |
| 119 | 2 | Satt459 | | 118.62 | | P | |  | 163 | 13 | Satt325 | | 2.23 | P | |
| 120 | 2 | Satt266 | | 59.61 | | P | |  | 164 | 13 | Sat_039 | | 27.87 | M | |
| 121 | 2 | BE475335 | | 30.74 | | P | |  | 165 | 13 | Satt_390 | | 1.79 | M | |
| 122 | 2 | Satt282 | | 76.10 | | M | |  | 166 | 13 | Sat_317 | | 72.97 | P | |
| 123 | 2 | Sat_351 | | 20.61 | | M | |  | 167 | 13 | Sat_113 | | 50.78 | M | |
| 124 | 2 | AI856415 | | 50.11 | | M | |  | 168 | 13 | Satt663 | | 56.17 | M | |
| 125 | 2 | Sat_254 | | 46.92 | | M | |  | 169 | 13 | Satt114 | | 63.69 | P | |
| 126 | 2 | BE070293 | | 47.28 | | M | |  | 170 | 13 | Sat_313 | | 91.87 | M | |
| 127 | 2 | Satt290 | | 73.35 | | M | |  | 171 | 18 | Sat_403 | | 34.87 | P | |
| 128 | 2 | Satt192 | | 44.04 | | M | |  | 172 | 18 | Sat_210 | | 3.70 | M | |
| 129 | 2 | Satt157 | | 37.07 | | M | |  | 173 | 18 | Sat_141 | | 9.18 | M | |
| 130 | 2 | Satt141 | | 72.89 | | M | |  | 174 | 18 | Satt038 | | 1.84 | P | |

Contd....

| **Sl.No** | **Chrom** | **SSRmarker** | **Distance(cM)** | **P/M** |  | **Sl.No** | **Chrom** | **SSR marker** | **Distance(cM)** | **P/M** |
| --- | --- | --- | --- | --- | --- | --- | --- | --- | --- | --- |
| 175 | 18 | Sat_131 | 31.33 | M |  | 219 | 16 | Sat_255 | 43.85 | P |
| 176 | 18 | Sat_094 | 56.18 | M |  | 220 | 16 | Satt183 | 42.51 | P |
| 177 | 18 | Satt012 | 66.55 | M |  | 221 | 16 | Sat_224 | 75.13 | P |
| 178 | 18 | Sat_064 | 108.74 | M |  | 222 | 16 | Satt529 | 41.90 | M |
| 179 | 18 | Satt130 | 23.1 | M |  | 223 | 16 | Satt674 | 15.95 | P |
| 180 | 18 | Sat_168 | 3.90 | M |  | 224 | 16 | Sat_394 | 89.43 | M |
| 181 | 18 | Satt517 | 69.87 | P |  | 225 | 16 | Satt215 | 44.08 | P |
| 182 | 18 | Sat_143 | 73.42 | M |  | 226 | 9 | Sat_087 | 4.85 | P |
| 183 | 18 | AW734137 | 15.63 | P |  | 227 | 9 | Satt240 | 52.88 | M |
| 184 | 18 | Satt191 | 96.57 | M |  | 228 | 9 | Satt247 | 43.96 | M |
| 185 | 12 | Sat_175 | 83.19 | P |  | 229 | 9 | Satt381 | 44.99 | P |
| 186 | 12 | Satt666 | 0.59 | M |  | 230 | 9 | Sat_349 | 50.39 | M |
| 187 | 12 | Sctt009 | 38.89 | M |  | 231 | 9 | Satt196 | 104.79 | M |
| 188 | 12 | Satt442 | 46.95 | M |  | 232 | 9 | Satt260 | 80.12 | P |
| 189 | 12 | Satt222 | 68.08 | M |  | 233 | 9 | Sat_116 | 52.28 | M |
| 190 | 12 | Sat_218 | 99.5 | M |  | 234 | 9 | Satt337 | 47.38 | M |
| 191 | 12 | Satt676 | 68.86 | P |  | 235 | 9 | Sat_352 | 93.64 | M |
| 192 | 12 | Satt253 | 67.17 | M |  | 236 | 9 | Satt178 | 40.86 | M |
| 193 | 12 | Satt314 | 69.12 | M |  | 237 | 9 | Sat_243 | 86.78 | P |
| 194 | 12 | Sat_180 | 104.37 | M |  | 238 | 9 | Satt588 | 117.08 | P |
| 195 | 12 | Sat_158 | 73.46 | M |  | 239 | 19 | Satt481 | 54.87 | P |
| 196 | 12 | Sat_214 | 2.85 | P |  | 240 | 19 | Sat_245 | 115.07 | M |
| 197 | 12 | Satt279 | 68.50 | M |  | 241 | 19 | Sat_113 | 68.26 | M |
| 198 | 12 | Sat_205 | 68.18 | P |  | 242 | 19 | Satt232 | 10.35 | M |
| 199 | 12 | Satt302 | 81.04 | M |  | 243 | 19 | Satt664 | 92.66 | M |
| 200 | 20 | Sat_324 | 84.48 | M |  | 244 | 19 | Sat_301 | 11.12 | M |
| 201 | 20 | Satt451 | 20.34 | P |  | 245 | 19 | Sat_245 | 115.08 | P |
| 202 | 20 | Satt049 | 58.82 | P |  | 246 | 19 | Sat_099 | 78.23 | M |
| 203 | 20 | Satt700 | 35.03 | P |  | 247 | 19 | Satt418 | 30.93 | P |
| 204 | 20 | Sat_155 | 98.06 | M |  | 248 | 19 | Sat_286 | 87.42 | M |
| 205 | 20 | Sat_104 | 65.62 | M |  | 249 | 19 | Sat_408 | 1.31 | P |
| 206 | 20 | Sat_650 | 63.33 | M |  | 250 | 19 | Satt006 | 92 | M |
| 207 | 20 | Sct_189 | 113.77 | P |  | 251 | 7 | Sat_389 | 0 | P |
| 208 | 20 | Sat_299 | 99.83 | M |  | 252 | 7 | Satt567 | 33.47 | M |
| 209 | 20 | Sat_390 | 1.79 | M |  | 253 | 7 | Satt404 | 0.84 | M |
| 210 | 20 | Satt623 | 92.52 | M |  | 254 | 7 | Satt435 | 38.94 | P |
| 211 | 20 | Satt330 | 77.84 | M |  | 255 | 7 | Satt201 | 13.56 | M |
| 212 | 20 | Satt292 | 82.78 | M |  | 256 | 7 | Satt618 | 111.06 | M |
| 213 | 20 | Satt239 | 36.94 | M |  | 257 | 7 | Sat_316 | 21 | M |
| 214 | 16 | Satt285 | 25.51 | P |  | 258 | 7 | Satt336 | 133.83 | P |
| 215 | 16 | Satt405 | 12.41 | M |  | 259 | 7 | Sat_244 | 48.36 | M |
| 216 | 16 | Sat_366 | 52.84 | P |  | 260 | 7 | Satt494 | 71.71 | M |
| 217 | 16 | Satt620 | 53.71 | M |  | 261 | 7 | Sat_144 | 122.37 | M |
| 218 | 16 | Satt693 | 33.88 | M |  | 262 | 7 | Satt308 | 130.76 | P |

Contd...

| **Sl.No** | **Chrom** | **SSR marker** | **Distance(cM)** | **P/M** |
| --- | --- | --- | --- | --- |
| 263 | 3 | Satt009 | 28.52 | P |
| 264 | 3 | Sat_084 | 38.86 | M |
| 265 | 3 | Satt530 | 32.85 | M |
| 266 | 3 | Sct_195 | 2.44 | P |
| 267 | 3 | Sat_033 | 58.38 | M |
| 268 | 3 | Sat_304 | 77.10 | M |
| 269 | 3 | Sat_125 | 103.34 | M |
| 270 | 3 | Sat_186 | 30.11 | M |
| 271 | 3 | Stt022 | 102.6 | M |
| 272 | 3 | Satt257 | 92.56 | M |
| 273 | 3 | Sat_036 | 93.11 | M |
| 274 | 3 | Satt624 | 35.32 | P |
| 275 | 3 | Satt312 | 79.86 | M |
| 276 | 3 | Satt549 | 70.60 | P |
| 277 | 3 | Sat_295 | 95.00 | M |
| 278 | 10 | Sat_190 | 129.8 | P |
| 279 | 10 | Satt492 | 17.25 | M |
| 280 | 10 | Satt487 | 9.53 | M |
| 281 | 10 | Sat_282 | 63.81 | P |
| 282 | 10 | Satt500 | 14.17 | M |
| 283 | 10 | Satt653 | 38.09 | P |
| 284 | 10 | Sat_303 | 20.93 | M |
| 285 | 10 | Satt347 | 42.29 | M |
| 286 | 10 | Satt592 | 100.38 | M |
| 287 | 10 | Satt331 | 93.37 | M |
| 288 | 10 | Satt581 | 106.03 | M |
| 289 | 10 | Sat_108 | 129.30 | M |
| 290 | 10 | Satt173 | 58.40 | P |

* Chrom: Chromosome number; P: Polymorphic; M: Monomorphic

Table S7 **Details of the Simple Sequence Repeat markers screened for parental polymorphism between the recurrent (DS9814) and donor parent (PI542044)**

| **Sl. No.** | **Linkage Group** | **Chromosome. No.** | **SSR Marker** | **Distance**  **(cM)** | **P/M** | |
| --- | --- | --- | --- | --- | --- | --- |
| 1 | A1 | 5 | Sat_368 | 14.37 | | M |
| 2 | A1 | 5 | Satt385 | 64.74 | | M |
| 3 | A1 | 5 | Satt511 | 94.20 | | M |
| 4 | A1 | 5 | Satt545 | 71.39 | | M |
| 5 | A1 | 5 | Satt599 | 85.58 | | P |
| 6 | A1 | 5 | Satt619 | 69.21 | | M |
| 7 | A1 | 5 | Satt717 | 51.95 | | P |
| 8 | A1 | 5 | Sat_267 | 78.45 | | M |
| 9 | A1 | 5 | Satt175 | 66.99 | | M |
| 10 | A1 | 5 | Sat_271 | 97.76 | | M |
| 11 | A1 | 5 | Satt200 | 92.89 | | P |
| 12 | A1 | 5 | Satt174 | 88.58 | | P |
| 13 | A1 | 5 | Satt593 | 25.56 | | M |
| 14 | A1 | 5 | Satt648 | 59.18 | | M |
| 15 | A1 | 5 | Satt211 | 95.96 | | M |
| 16 | A1 | 5 | Satt050 | 46.45 | | M |
| 17 | A2 | 8 | Satt177 | 36.77 | | M |
| 18 | A2 | 8 | Sat_199 | 84.09 | | P |
| 19 | A2 | 8 | Sct_067 | 14.99 | | M |
| 20 | A2 | 8 | Sat_215 | 53.75 | | P |
| 21 | A2 | 8 | Sat_181 | 38.06 | | P |
| 22 | A2 | 8 | Satt228 | 154.11 | | P |
| 23 | A2 | 8 | Sat_347 | 158.39 | | P |
| 24 | A2 | 8 | Sat_377 | 116.64 | | M |
| 25 | A2 | 8 | Satt538 | 159.63 | | M |
| 26 | A2 | 8 | Satt429 | 162.03 | | M |
| 27 | A2 | 8 | Satt409 | 145.57 | | P |
| 28 | A2 | 8 | GMENOD2B | 58.44 | | M |
| 29 | B1 | 11 | Sat_095 | 81.31 | | M |
| 30 | B1 | 11 | Sat_156 | 35.00 | | P |
| 31 | B1 | 11 | Sat_149 | 54.01 | | P |
| 32 | B1 | 11 | Satt665 | 96.36 | | P |
| 33 | B1 | 11 | Satt453 | 123.96 | | M |
| 34 | B1 | 11 | Satt197 | 46.39 | | M |
| 35 | B1 | 11 | Satt415 | 82.89 | | M |

**Contd...**

| **Sl. No.** | **Linkage Group** | **Chromosome. No.** | **SSR Marker** | **Distance**  **(cM)** | **P/M** |
| --- | --- | --- | --- | --- | --- |
| 36 | B1 | 11 | Sat_411 | 30.87 | M |
| 37 | B2 | 14 | Satt560 | 97.92 | M |
| 38 | B2 | 14 | Satt534 | 87.59 | M |
| 39 | B2 | 14 | Satt467 | 17.77 | M |
| 40 | B2 | 14 | Satt070 | 72.81 | P |
| 41 | B2 | 14 | Satt556 | 73.21 | P |
| 42 | B2 | 14 | Sat_287 | 31.88 | P |
| 43 | B2 | 14 | Satt168 | 55.20 | M |
| 44 | B2 | 14 | Sat_230 | 72.08 | M |
| 45 | B2 | 14 | Satt687 | 113.61 | M |
| 46 | C1 | 4 | Sat_367 | 28.04 | P |
| 47 | C1 | 4 | AW277661 | 74.79 | P |
| 48 | C1 | 4 | Satt565 | 0.00 | P |
| 49 | C1 | 4 | Satt396 | 24.11 | M |
| 50 | C1 | 4 | Sct_186 | 9.02 | M |
| 51 | C1 | 4 | Satt361 | 75.52 | M |
| 52 | C1 | 4 | Satt164 | 132.46 | M |
| 53 | C1 | 4 | Satt690 | 5.36 | M |
| 54 | C1 | 4 | Sat_322 | 79.27 | P |
| 55 | C1 | 4 | Satt180 | 127.77 | M |
| 56 | C2 | 6 | Satt460 | 117.77 | P |
| 57 | C2 | 6 | Satt643 | 94.65 | P |
| 58 | C2 | 6 | Sat_238 | 117.46 | P |
| 59 | C2 | 6 | Satt277 | 107.59 | P |
| 60 | C2 | 6 | AW734043 | 4.22 | M |
| 61 | C2 | 6 | Satt202 | 126.24 | P |
| 62 | C2 | 6 | Satt286 | 101.75 | M |
| 63 | C2 | 6 | Satt170 | 70.56 | M |
| 64 | C2 | 6 | Satt640 | 30.47 | M |
| 65 | D1a | 1 | Satt179 | 56.20 | P |
| 66 | D1a | 1 | Sat_413 | 5.93 | M |
| 67 | D1a | 1 | Sat_036 | 75.25 | M |
| 68 | D1a | 1 | Satt507 | 64.52 | P |
| 69 | D1a | 1 | Satt408 | 106.69 | M |
| 70 | D1a | 1 | Satt531 | 40.87 | M |
| 71 | D1a | 1 | Satt407 | 99.59 | M |
| 72 | D1a | 1 | Satt532 | 49.07 | M |

**Contd...**

| **Sl. No.** | **Linkage Group** | **Chromosome No.** | **SSR Marker** | **Distance**  **(cM)** | **P/M** |
| --- | --- | --- | --- | --- | --- |
| 73 | D1a | 1 | Satt342 | 48.14 | M |
| 74 | D1a | 1 | Satt370 | 60.99 | M |
| 75 | D1b | 2 | Satt459 | 118.62 | P |
| 76 | D1b | 2 | Satt266 | 59.61 | P |
| 77 | D1b | 2 | Satt542 | 53.02 | M |
| 78 | D1b | 2 | BE475343 | 30.74 | P |
| 79 | D1b | 2 | Satt282 | 76.10 | M |
| 80 | D1b | 2 | Satt290 | 73.35 | P |
| 81 | D1b | 2 | Satt157 | 37.07 | M |
| 82 | D1b | 2 | Satt542 | 53.02 | M |
| 83 | D1b | 2 | Satt296 | 52.61 | M |
| 84 | D1b | 2 | Satt558 | 43.91 | M |
| 85 | D1b | 2 | BF070293 | 47.28 | M |
| 86 | D1b | 2 | Sat_289 | 131.92 | M |
| 87 | D1b | 2 | Sat_211 | 38.04 | M |
| 88 | D2 | 17 | Satt311 | 84.62 | M |
| 89 | D2 | 17 | Satt186 | 105.45 | P |
| 90 | D2 | 17 | Sat_001 | 92.12 | P |
| 91 | D2 | 17 | Satt528 | 86.34 | P |
| 92 | D2 | 17 | Sat_220 | 128.73 | M |
| 93 | D2 | 17 | Satt662 | 87.88 | P |
| 94 | D2 | 17 | Sat_365 | 87.39 | M |
| 95 | D2 | 17 | GMHSP179 | 99.04 | M |
| 96 | E | 15 | Satt685 | 56.70 | M |
| 97 | E | 15 | Satt720 | 20.80 | M |
| 98 | E | 15 | Satt212 | 32.27 | M |
| 99 | E | 15 | Satt691 | 19.70 | P |
| 100 | E | 15 | Satt230 | 71.31 | M |
| 101 | E | 15 | Sat_112 | 8.67 | M |
| 102 | E | 15 | Satt381 | 44.99 | P |
| 103 | E | 15 | Satt213 | 3.72 | M |
| 104 | F | 13 | Satt114 | 63.69 | M |
| 105 | F | 13 | Satt656 | 135.12 | P |
| 106 | F | 13 | Satt252 | 16.08 | P |
| 107 | F | 13 | Sct_188 | 85.33 | P |
| 108 | F | 13 | Sat_317 | 72.97 | P |
| 109 | F | 13 | Satt663 | 56.17 | M |

**Contd...**

| **Sl. No.** | **Linkage Group** | **Chromosome No.** | **SSR Marker** | **Distance**  **(cM)** | **P/M** |
| --- | --- | --- | --- | --- | --- |
| 110 | F | 13 | Sat_313 | 91.87 | M |
| 111 | F | 13 | Sat_390 | 1.79 | M |
| 112 | G | 18 | Satt038 | 1.84 | M |
| 113 | G | 18 | Sat_403 | 34.87 | P |
| 114 | G | 18 | Satt517 | 69.87 | P |
| 115 | G | 18 | AW734137 | 15.63 | P |
| 116 | G | 18 | Satt130 | 23.10 | M |
| 117 | H | 12 | U08405 | 7.26 | P |
| 118 | H | 12 | Sat_175 | 83.19 | M |
| 119 | H | 12 | Sat_205 | 68.18 | P |
| 120 | H | 12 | Satt302 | 81.04 | M |
| 121 | H | 12 | Satt666 | 0.59 | P |
| 122 | H | 12 | Satt142 | 86.49 | M |
| 123 | H | 12 | Satt676 | 68.86 | P |
| 124 | H | 12 | Sat_214 | 2.85 | P |
| 125 | H | 12 | Satt353 | 8.48 | P |
| 126 | I | 20 | Sat_155 | 98.06 | P |
| 127 | I | 20 | Satt623 | 92.52 | P |
| 128 | I | 20 | Satt330 | 77.84 | M |
| 129 | I | 20 | Satt292 | 82.78 | P |
| 130 | I | 20 | Satt451 | 20.34 | P |
| 131 | I | 20 | Satt049 | 58.82 | P |
| 132 | I | 20 | Sct_189 | 113.77 | P |
| 133 | I | 20 | Sat_299 | 99.83 | M |
| 134 | I | 20 | Sat_104 | 65.62 | M |
| 135 | J | 16 | Sat_394 | 89.43 | M |
| 136 | J | 16 | Satt215 | 44.08 | P |
| 137 | J | 16 | Satt285 | 25.51 | P |
| 138 | J | 16 | Sat_366 | 52.84 | P |
| 139 | J | 16 | Satt674 | 15.95 | P |
| 140 | J | 16 | Satt183 | 42.51 | P |
| 141 | J | 16 | Sat_224 | 75.13 | M |
| 142 | J | 16 | Sat_255 | 43.85 | M |
| 143 | K | 9 | Sat_243 | 86.78 | P |
| 144 | K | 9 | Satt381 | 44.99 | P |
| 145 | K | 9 | Satt260 | 80.12 | P |
| 146 | K | 9 | Sat_352 | 93.64 | P |

**Contd...**

| **Sl. No.** | **Linkage Group** | **Chromosome No.** | **SSR Marker** | **Distance**  **(cM)** | **P/M** |
| --- | --- | --- | --- | --- | --- |
| 147 | K | 9 | Sat_087 | 4.85 | M |
| 148 | K | 9 | Satt588 | 117.02 | P |
| 149 | K | 9 | Satt178 | 40.86 | M |
| 150 | K | 9 | Satt196 | 104.79 | M |
| 151 | K | 9 | Satt337 | 47.38 | M |
| 152 | L | 19 | Sat_408 | 1.31 | P |
| 153 | L | 19 | Satt481 | 54.57 | M |
| 154 | L | 19 | Satt418 | 30.93 | P |
| 155 | L | 19 | Sat_245 | 115.07 | M |
| 156 | L | 19 | Sat_113 | 68.26 | P |
| 157 | L | 19 | Sat_286 | 87.42 | M |
| 158 | M | 7 | Sat_226 | 65.79 | M |
| 159 | M | 7 | Satt336 | 133.80 | P |
| 160 | M | 7 | Satt435 | 38.94 | M |
| 161 | M | 7 | Sat_389 | 0.00 | P |
| 162 | M | 7 | Satt308 | 130.70 | P |
| 163 | M | 7 | Satt494 | 71.71 | M |
| 164 | M | 7 | Satt567 | 33.47 | M |
| 165 | M | 7 | Satt618 | 111.06 | M |
| 166 | N | 3 | Satt549 | 70.60 | P |
| 167 | N | 3 | Satt022 | 102.06 | P |
| 168 | N | 3 | Satt009 | 28.52 | P |
| 169 | N | 3 | Satt624 | 35.32 | P |
| 170 | N | 3 | Satt530 | 32.85 | M |
| 171 | N | 3 | Sat_186 | 30.11 | M |
| 172 | N | 3 | Satt257 | 92.56 | M |
| 173 | O | 10 | Sat_190 | 129.80 | P |
| 174 | O | 10 | Satt173 | 58.40 | P |
| 175 | O | 10 | Satt653 | 38.09 | P |
| 176 | O | 10 | Sat_282 | 63.81 | P |
| 173 | O | 10 | Sat_190 | 129.80 | P |
| 174 | O | 10 | Satt173 | 58.40 | P |
| 175 | O | 10 | Satt653 | 38.09 | P |
| 176 | O | 10 | Sat_282 | 63.81 | P |
| 177 | O | 10 | Satt581 | 106.03 | M |
| 178 | O | 10 | Satt347 | 42.29 | M |
| 179 | O | 10 | Satt331 | 93.37 | M |
| 180 | O | 10 | Satt492 | 17.25 | M |

P: Polymorphic; M: Monomorphic
